# DNA sonification for public engagement in bioinformatics

**DOI:** 10.1186/s13104-021-05685-7

**Published:** 2021-07-15

**Authors:** Heleen Plaisier, Thomas R. Meagher, Daniel Barker

**Affiliations:** 1 Institute of Evolutionary Biology, School of Biological Sciences, University of Edinburgh, Charlotte Auerbach Road, The King’s Buildings, Edinburgh, EH9 3FL UK; 2 Present Address: Royal Botanic Garden Edinburgh, 20A Inverleith Row, Edinburgh, EH3 5LR UK; 3 Centre for Biological Diversity, School of Biology, University of St Andrews, Sir Harold Mitchell Building, Greenside Place, St Andrews, KY16 9TH UK

**Keywords:** Bioinformatics, Sonification, DNA, Sequence, Public engagement, Raspberry Pi, Sonic Pi

## Abstract

**Objective:**

Visualisation methods, primarily color-coded representation of sequence data, have been a predominant means of representation of DNA data. Algorithmic conversion of DNA sequence data to sound—sonification—represents an alternative means of representation that uses a different range of human sensory perception. We propose that sonification has value for public engagement with DNA sequence information because it has potential to be entertaining as well as informative. We conduct preliminary work to explore the potential of DNA sequence sonification in public engagement with bioinformatics. We apply a simple sonification technique for DNA, in which each DNA base is represented by a specific note. Additionally, a beat may be added to indicate codon boundaries or for musical effect. We report a brief analysis from public engagement events we conducted that featured this method of sonification.

**Results:**

We report on use of DNA sequence sonification at two public events. Sonification has potential in public engagement with bioinformatics, both as a means of data representation and as a means to attract audience to a drop-in stand. We also discuss further directions for research on integration of sonification into bioinformatics public engagement and education.

## Introduction

As the field of genomics has matured, the need to provide novel tools for representing DNA sequence data has become pressing. This need ranges from comparative investigation of sequence variation across multiple sequences, searches for functional domains across extended sequences, searches for sequences within and between organisms that share homology or are otherwise similar, and capturing and comparing entire genomes. As genomics data and its potential for scientific investigation has grown, so too have methods for representing data. Visual sequence data has been a standard approach, but it has its limitations, especially when long sequences are involved. Algorithmic conversion of DNA sequences to sound—sonification—offers an alternative means of representing DNA sequences that in turn draws upon other human sensory mechanisms. Sonification may have very general value, though the field of bioinformatics sonification is not yet sufficiently mature to support (or reject) this as a broad conclusion.

DNA sequence visualisation has a relatively long history in bioinformatics. Even the representation of the four bases as letters in the alphabet—A, C, G and T, with the sequence of one strand of the double helix represented as a string of such letters—is a simple visualisation, with a long tradition in databases and publications involving DNA sequence (e.g., [1, 2]). Visualisation of genome annotation, pairwise sequence alignment diagrams and multiple alignments take visualisation further, by adding visualisation of properties of the sequence (e.g., predicted gene structures or similarity to other sequences).

However, like any representation, visualisation has limits. In a research context, visualisation of large amounts of sequence is constrained by the size of a computer screen, the ability to convey diverse information through colours or symbols, and the attention of the researcher. In the context of public engagement with science, DNA sequence has a monotonous visual appearance, at odds with the “whizz-bang” often expected in such public displays or shows.

Although the quantity and apparently random nature of large DNA sequences is engaging for a short time, more detailed examination of DNA sequence or annotation demands a level of focus that is atypically high for a public drop-in event or short activity. At public events we have had success with a wide range of ages using the Phylo game [3], where anyone can attempt to improve multiple alignments of regions of genes associated with human disease [4]. However, any one game or activity can only present a small aspect of bioinformatics. Although we have had success introducing bioinformatics to school pupils [5, 6] and introducing bioinformatics and computational science to the public in a science centre [4], bioinformatics as a whole is rather lacking in public engagement activities.

We aim to help fill this gap by means of sonification of DNA, where the representation of sequence is auditory rather than visual. Since music is widely understood and used for relaxation and entertainment, this may be more attractive to a public audience than a screen full of DNA sequence or associated data. By use of the Sonic Pi program [7] for sonification, we make the underlying program visible and customisable in real-time. This also provides a link between DNA and programming, which in is central to bioinformatics in general.

Sonification of scientific data in is not new (e.g., [8]). More specifically, the use of DNA sequence to generate music has a history in music composition for artistic purposes, and there is a developing literature on DNA sonification for research (e.g., [9–11]). However, though DNA sonification examples for public engagement exist (e.g., [10, 12, 13]), they appear rare. Based on our preliminary activities, DNA sonification has potential for successful public engagement activities. Further efforts in this area are warranted. We report a brief analysis from public engagement events we have conducted that featured sonification. We also discuss some further directions for research on integration of sonification into public engagement.

## Main text

### Methods

At Picademy in Glasgow on 29 November 2016, an initial sonification project was carried out. The coding sequence of the l-gulonolactone oxidase gene from mouse (*Gulo*; as is used in our separate workshops for school classes [14, 15]) was converted to a Sonic Pi program, using search-and-replace to convert bases A, C, G and T respectively to notes A4 or note 69 in the Musical Instrument Digital Interface (MIDI) standard, C4 (MIDI 60), G4 (MIDI 67) and G#0 (MIDI 20, chosen just because T is 20th letter in the alphabet), using the default synthesiser. A filtered bass drum was added at the start of each codon (i.e., every three bases). Subsequent events used Raspberry Pi computers running the 4273pi variant of GNU/Linux [16]. 4273pi comes with Sonic Pi installed. Sequence and script files—for example from our Additional files—can be transferred to the Pi via a Universal Serial Bus (USB) stick.

Subsequently we incorporated sonification into a series of public events as follows.

In preparation for Doors Open Day, a public drop-in event where we were based at the Ashworth Laboratories, King’s Buildings, University of Edinburgh on 23 Sept, 2017 from 10 am to 4 pm [17], further sonifications were prepared, using a Perl script we wrote to convert DNA sequence in Fasta format to a Sonic Pi program followed by manual editing of the result. For script and example Sonic Pi programs and sound files, see Additional files. The event brought in a range of people, some potential new students and parents who were interested in biology, but also families with young children and other local people. For this event, the sonification of the base T was changed from MIDI 20, which Sonic Pi can play but is difficult to hear, to MIDI 64 (E4). These additional sonifications consisted of: (1) a codon-free version of *Gulo*; (2) a rave version of the *Gulo* sonification, using the “mod_saw” synthesizer, with a drum roll starting every 4th note for musical effect; and (3) a sonification of part of an intron of the CF transmembrane conductance regulator gene (*CFTR*) in human, which includes a dinucleotide microsatellite (AT), to highlight the potential research benefit of sonification (using no drums). Tempos were from 60 equal-length notes per minute up to 300 notes per minute, set using a time parameter to the “play_pattern_timed” function. Additionally, a brief tutorial to Sonic Pi including DNA sonification exercises was written and printed out in laminated form, along with the *Gulo* coding sequence and the start of the intron sequence from *CFTR*, with the microsatellites highlighted. Doors Open Day 2017 at the Ashworth Laboratories was attended by at least 258 people. During the event, we engaged ~ 30 visitors in a discussion about DNA, mainly by approaching them with the question “Have you ever wondered what DNA would sound like?”. The event was evaluated by means of direct experience and diaries of H.P. and D.B.

As part of Ada Lovelace Day, in a free but ticketed public event at the James Clerk Maxwell Building, King’s Buildings, University of Edinburgh on 10 October 2017 [18], we gave an introductory presentation for ~ 50 participants, and offered three workshops later in the day, which were attended by a total of 13 people. The workshops lasted approximately 30–40 min each and consisted of a short DNA investigation using BLAST [19] with the *Gulo* gene (as in [15]), followed by a guided exploration of prepared DNA sonifications, and the opportunity to go through the same printed sonification tutorial. The event was evaluated by means of a short questionnaire and diaries of H.P. and D.B.

### Results and discussion

Our preliminary study indicates that DNA sonification is highly suitable for public engagement activities, both for short drop-in events and for more focused workshops.

Doors Open Day was a large public event, during which people engaged well with the sonification activity. The question “Have you ever wondered what DNA would sound like” was a successful way of starting a discussion about DNA. Everyone who was asked this question stayed to listen to a sample of our sonifications and engaged in a short discussion about DNA. Visitors were guided through prepared sonifications, and given the opportunity to go through the printed sonification tutorial. Visitors found the event creative and inspiring and were either happy to see it as a light distraction, or to discuss scientific applications. Relatively little use was made of the Sonic Pi tutorial. During the event, Minecraft proved a distraction, so we discreetly removed it from the main menu of each Raspberry Pi. Later, Scratch was also some distraction. For the future, we would remove these in advance because they are not part of our display. Restrictions on Web browser access based on Uniform Resource Locator (URL) may also have some value. The Phylo game ran slowly at times on the Raspberry Pi computers (a problem that would be reduced or removed with newer models of the hardware).

The sonification workshops during Ada Lovelace Day were well-attended, and the audience appeared engaged. Six participants completed questionnaires. Others did not, due to leaving early for lunch (1 participant) and leaving at short notice to collect their bags from the main activity room (5 participants). When asked what the best thing about the workshop was, participants stated “The opportunity to engage with DNA or Biology from a different perspective. Hearing a sequence made me think of it as an object in a different way.”; “Sonic Pi (amazing!)”; and “Updating me on sequence investigation”. When asked what the worst thing about the workshop was, participants did not have many comments, i.e., “None”, “Nothing”, “not enough time!”. We had difficulty connecting to the University wireless network and used a 4G phone as a hotspot instead. This was resolved before participants arrived and was not noticeable to them. The event was a success. However, H.P. and D.B. thought a little more time to focus on sonification would be helpful for such events in the future.

It appears that DNA sonification engages both researchers and the public in thinking about DNA from a different perspective. Further development of DNA sonification for public engagement activities is warranted.

## Limitations

Although our preliminary study is sufficient to suggest DNA sonification has potential in public engagement activities, a larger study is required to discover the full limits of its successful application. Our audience for evaluation consisted of adults, mostly staff and students at Edinburgh University. We recommend a larger programme, using qualitative and quantitative analysis, with a diverse range of audiences.

We regard our simple sonification technique—using one note per base—as more relevant to public engagement than to research. The limited range of notes makes for a catchy melody but it is difficult to distinguish sequence features, beyond simple examples such as a long dinucleotide repeat. However, for public engagement, it is important to avoid a strong reliance on biological knowledge.

For research, future directions involve sonification of more complex bioinformatics data, for example multiple sequence alignments (Martin et al., in prep.). Multiple alignment depends on biological concepts such as homology and cross-sequence comparison, unlikely to be rapidly comprehensible to the general public. The Phylo multiple alignment game [3] is a counter-example. However, as well as an exercise in multiple alignment, Phylo may be understood as a logic puzzle, potentially bypassing biological concepts for many users while leaving them as useful discussion points for those with expertise in the area.

For public engagement and education, one promising future direction may be to highlight the effect of frameshift mutations. We have developed and apply a workshop for biology students in secondary education [15] (open educational resources available at [14]), centred on the *Gulo* gene (e.g., [20]), which in humans is disrupted by frameshift mutations. Visually, a frameshift mutation is difficult to notice on-screen in BLAST alignments comparing the *Gulo* coding sequence and the human pseudogene. A frameshift is often only indicated by a single dash (“-”) among DNA symbols. Sonification could help make the frameshift stand out.

In conclusion, sonification has a demonstrated potential for public outreach and engagement. Our methodology was well-received. It is our aspiration to build on these preliminary efforts to use sonification to make DNA sequence information entertaining as well as informative and to increase the nuance and complexity that we convey in public engagement.

## Data Availability

Sound files, a Perl script, Sonic Pi scripts and a tutorial supporting the conclusions of this article are available in the University of Edinburgh’s DataShare repository, https://doi.org/10.7488/ds/3058.

## Abbreviations

*CFTR*: CF transmembrane conductance regulator gene
*Gulo*: l-Gulonolactone oxidase gene
MIDI: Musical instrument digital interface
URL: Uniform resource locator
USB: Universal serial bus
